# Seasonal Changes in the Distinct Taxonomy and Function of the Gut Microbiota in the Wild Ground Squirrel (*Spermophilus dauricus*)

**DOI:** 10.3390/ani11092685

**Published:** 2021-09-13

**Authors:** Xiaoying Yang, Yuchen Yao, Xueying Zhang, Jiahui Zhong, Fuli Gao, Haolin Zhang, Yingying Han, Qiang Weng, Zhengrong Yuan

**Affiliations:** College of Biological Sciences and Technology, Beijing Forestry University, Beijing 100083, China; xiaoying_yang@bjfu.edu.cn (X.Y.); charming_yao@outlook.com (Y.Y.); wangzai619@bjfu.edu.cn (X.Z.); zhongjiahui@bjfu.edu.cn (J.Z.); fuligao@bjfu.edu.cn (F.G.); haolinzhang@bjfu.edu.cn (H.Z.); thinkinghyy@bjfu.edu.cn (Y.H.); qiangweng@bjfu.edu.cn (Q.W.)

**Keywords:** seasonal breeding, gut microbiota, wild ground squirrel, 16S rRNA sequencing, energy metabolism

## Abstract

**Simple Summary:**

Gut microbiota is a large number of microbes colonized in the gut tract, and it plays a certain role in regulating the host’s immunity, metabolism, and nervous system. Recent studies have shown that the gut microbiota also has a close relationship with reproduction. The wild ground squirrel (*Spermophilus dauricus*) is a typical seasonal breeding animal. The purpose of this study was to explore the distinct taxonomy and function of the gut microbiota in the breeding and non-breeding seasons of the wild ground squirrel using 16S rRNA gene sequencing technology. The results show that the taxonomy of gut microbiota was different between the breeding season and non-breeding season. Functional prediction of the gut microbiota indicated that the relative abundance of metabolic pathways was differentially enriched between the breeding season and non-breeding season. This study further revealed the potential relationship between gut microbiota and reproduction and expanded our understanding of the function of gut microbiota. At the same time, it provided a new direction for research on the breeding strategy of seasonal breeding animals.

**Abstract:**

Seasonal breeding is a normal phenomenon in which animals adapt to natural selection and reproduce only in specific seasons. Large studies have reported that the gut microbiota is closely related to reproduction. The purpose of this study was to explore the distinct taxonomy and function of the gut microbiota in the breeding and non-breeding seasons of the wild ground squirrel (*Spermophilus dauricus*). The 16S rRNA gene sequencing technology was utilized to sequence the gut microbiota of the wild ground squirrel. PICRUSt analysis was also applied to predict the function of the gut microbiota. The results suggested that the main components of the gut microbiota in all samples were Firmicutes (61.8%), Bacteroidetes (32.4%), and Proteobacteria (3.7%). Microbial community composition analyses revealed significant differences between the breeding and non-breeding seasons. At the genus level, *Alistipes*, *Mycoplasma*, *Anaerotruncus*, and *Odoribacter* were more abundant in the non-breeding season, while *Blautia* and *Streptococcus* were more abundant in the breeding season. The results of a functional prediction suggested that the relative abundance of functional categories that were related to lipid metabolism, carbohydrate metabolism, and nucleotide metabolism increased in the breeding season. The relative abundance of energy metabolism, transcription, and signal transduction increased in the non-breeding season. Overall, this study found differences in the taxonomy and function of the gut microbiota of the wild ground squirrel between the breeding and non-breeding seasons, and laid the foundation for further studies on the relationship between the gut microbiota and seasonal breeding.

## 1. Introduction

The gut microbiota of animals is composed of large numbers of microbes. The micropopulation stays in and interacts with the host, forming a balanced, complex, and diverse gut microbiota system [1]. The gut microbiota is closely related to the physiological activities and growth of the body and participates in many physiological processes, including metabolism and reproduction [2,3]. Many studies have demonstrated that the gut microbiota has essential effects on host growth [4], bone mineral density [5], energy metabolism [2], and immune regulation [6]. Furthermore, it has been found that the gut microbiota is closely linked to a variety of diseases, including obesity [7,8], diabetes [9], atherosclerosis [10], cancer [11], etc. The effects of the gut microbiota on reproduction have also received considerable attention. For example, studies on zebrafish (*Danio rerio*) illustrated that the probiotic *Lactobacillus rhamnosus* could activate leptin, which regulates the hypothalamus-pituitary-gonadal (HPG) axis to affect reproduction, thereby promoting the maturation of follicles and improving the reproductive ability of animals [3]. Moreover, in human beings, significant changes in gut microbial diversity occur during pregnancy, thus adjusting the metabolism to adapt to growing energy needs [12]. The gut microbiota also plays an important role in the synthesis, metabolism, and recycling of nitrogenous compounds such as amino acids. It has a significant influence on the host in terms of fecundity [13]. Therefore, the gut microbiota is crucial for the health and physiological activities of the host in terms of maintaining the health and ensuring the reproductive capacity of the host.

Seasonal breeding is a survival strategy so that animals can reproduce in the most favorable environments. Survival and reproduction are the primary tasks of each individual, and these tasks depend on their ability to adapt to seasonal variations and meet their own needs under the conditions of changes in food distribution, supply, and abundance [14]. It is well known that the reproductive function of mammals is mainly regulated by the HPG axis, which is activated only during the breeding season for seasonal breeders [15]. In recent years, it has been shown that the gut microbiota does interact with estrogens [16]. An estrobolome, the gene pool that can metabolize estrogen, exists in the gut microbiota [17]. The gut microbiota can affect the estrogen level by the secretion of β-glucuronidase, which can bind to estrogen receptor alpha (ERα) and estrogen receptor beta (ERβ) and affect downstream physiological effects [16]. However, whether the taxonomy and function of the gut microbiota are different in the breeding and non-breeding seasons is still unclear.

The wild ground squirrel (*Spermophilus dauricus*) is categorized into Mammalia, Rodentia, Sciuridae, and *Spermophilus*, and is a typical seasonal breeding small mammal. Wild ground squirrels breed only from April to May, which is called the breeding season, followed by the non-breeding season from June to the following March [18,19,20]. The wild ground squirrel is a suitable animal model for the study of seasonal breeding [21,22]. Our previous studies have shown that the android receptor (AR), estrogen receptors α and β (ER α, ER β), and aromatase cytochrome P450 (P450arom) are seasonally expressed in the hypothalamus, uterus, testes, and epididymis, which are the organs of the HPG axis [23,24,25,26]. Moreover, ghrelin, obestatin, insulin, and other gut-derived hormones have direct or indirect effects on the reproductive axis and play roles in regulating energy balance and reproductive function [27]. Previous studies have shown that the gut microbiota plays an important role in regulating the reproduction of the host [28,29]. However, there are no related studies that reported the association between the gut microbiota and seasonal breeding. Thus, this study aimed to explore the differences in the gut microbiota between the breeding and non-breeding seasons, and to further analyze the main functional differences.

## 2. Materials and Methods

### 2.1. Sample Collection

All wild ground squirrels were collected in the wild in Hebei Province, China. Six male individuals were captured in the breeding season (B; April; *n* = 6) and the non-breeding season (NB; June; *n* = 6), respectively. The cecal contents were collected quickly, and frozen in liquid nitrogen immediately, then stored at −80 °C before DNA extraction.

All animal experiments were approved by the Policy on the Care and Use of Animals by the Ethical Committee of Beijing Forestry University and the Department of Agriculture of Hebei Province, China (JNZF11/2007).

### 2.2. DNA Extraction and Sequencing

According to the instructions of the TIANamp DNA Kit (Tiangen Biotech Co., Ltd., Beijing, China), the total genomic DNA in each gut sample was extracted. The quality and integrity of DNA were evaluated by the A260/280 ratio and agarose gel electrophoresis. Then, the V3–V4 region of the bacteria 16S rRNA gene was amplified by two-step PCR, which used the forward primer 340F (5’-ACTCCTACGGGAGGCAGCAG-3’) and the reverse primer 806R (5’-GGACTACHVGGGTWTCTAAT-3’). All PCR reactions were carried out in 25 μL total volume that consisted of 12.5 μL KAPA HiFi HotStart ReadyMix (2×), 0.25 μmol/L of each primer, and 10 ng of DNA template. A KAPA HiFi Hotstart PCR Kit (KAPA Biosystems, United States) was used for the first round of PCR amplification. First, pre-denaturation was conducted at 95 °C for 3 min, then denaturation at 95 °C, annealing at 55 °C, and extension at 72 °C for 30 s. The three steps were repeated for 25 cycles and finally extended to 72 °C for 5 min. The PCR products were purified with 1× AMPure XP Beads, and then a second round PCR amplification was performed. Except for eight cycles, other cycle steps were the same as in the first round of PCR amplification. The PCR products of each sample were mixed to prepare the PCR amplicon libraries. Amplicons were extracted from 2% agarose gels and recycled using the QIAquick Gel Extraction Kit (Qiagen, Valencia, CA, USA), and then quantified with the KAPA Library Quantification Kit (KAPA Biosystems, Wilmington, MA, USA) as the standard of libraries mixing. After quality assessment and quantification, the same amounts of the amplified products were collected and sequenced by pair-end 2 × 300 bp in the Illumina Miseq platform from ORI-GENE Technology Co., Ltd. (Beijing, China).

### 2.3. Bioinformatic Analyses

High throughput sequencing results were received as the FASTQ file. Paired-end reads were merged with VSEARCH (v2.14.1, https://github.com/torognes/vsearch, accessed on 20 September 2020) [30] to form consensus sequences, and truncated at both primers. Afterward, reads with the error threshold above 0.01 were discarded. After removing low abundance noise with miniquesize 8, sequences were denoised to obtain single-base precision amplicon sequence variants (ASVs) using the UNOISE3 in USEARCH (v10.0.240, http://www.drive5.com/usearch/download.html, accessed on 20 September 2020) [31,32], meanwhile, chimeric sequences were also removed. To improve the accuracy, the Ribosomal Database Project (RDP) (training set 16, http://rdp.cme.msu.edu/, accessed on 27 May 2020) was applied as a reference sequence to remove chimeras again [33]. Furthermore, a feature table was created using USEARCH (v10.0.240) [34]. Subsequently, the taxonomic origin of each ASV was determined in VSEARCH (v2.14.1) with a confidence value of 0.60, based on the RDP (training set 16), and plastids and non-bacteria were removed.

After normalizing by subsample, the community richness index and community diversity index were calculated by the Vegan package [35] to determine the alpha diversity within the groups of the wild ground squirrel. Then, the number of ASVs in a 1–100% sequence without replacement was taken to calculate the richness change of the dilution process. Next, the beta diversity was calculated using the Bray-Curtis distance and visualized with Principal Coordinate Analysis (PCoA) to find the differences in microbiota structure between groups. Taxonomies can annotate species information from phylum to genus level. All the figures were generated using R software (v3.6.2, https://www.r-project.org/, accessed on 24 September 2020). Linear Discriminant Analysis Effect Size (LEfSe) was applied to analyze the differences at each level [36]. Based on the high-quality sequences, microbial functions were predicted by the Phylogenetic Investigation of Communities by Reconstruction of Unobserved States (PICRUSt) [37]. STAMP (v2.1.1.0, https://beikolab.cs.dal.ca/software/STAMP, accessed on 8 February 2020) [38] was used to explore the functional pathways with significant differences.

## 3. Results

### 3.1. Data Summary

A total of 1,812,298 16S rRNA gene reads were obtained from the raw data, with an average of 151,025 reads per sample. The raw data were merged at two ends, and clean sequences were obtained after quality control. The number of ASVs in each sample was obtained by denoising based on the clean sequences, eliminating the wrong sequences, and selecting credible sequences with higher abundance as the representative sequences. A total of 1635 ASVs were obtained from 12 samples. The average number of ASVs in the breeding season was 847 and that in the non-breeding season was 224 (Supplementary Materials Table S1). The raw data are available through the Genome Sequence Archive [39] in Beijing Institute of Genomics (BIG) Data Center (accession code CRA003793).

To evaluate whether the sequencing depth was enough to make a stable estimation of the species richness, we used the rarefaction curve, which approached saturation, indicating that sequencing was saturated and there was no need to increase the sample size. The sequencing depth covered all species in the samples, and the marginal contribution of more data to the discovery of new ASVs was very small (Supplementary Materials Figure S1).

### 3.2. Microbial Diversity

We analyzed Chao1, ACE, Shannon, and Simpson as the four common alpha diversity indexes of the gut microbiota (Supplementary Materials Table S2 and Figure S2). The alpha diversity index indicated that there was no significant difference in either the diversity index or the abundance index of the gut microbiota between the breeding season and non-breeding season.

**Figure 1:**
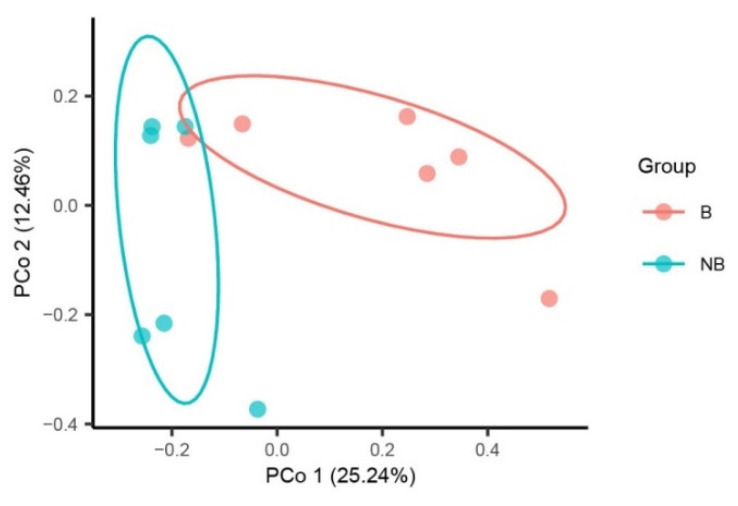
Beta diversity of the gut microbiota in the wild ground squirrel. Principal coordinate analysis (PCoA) plot of the gut microbiota structure using the Bray-Curtis distance metric. B, breeding season; NB, non-breeding season.

In terms of beta diversity, a PCoA analysis was carried out to determine the differences between the two groups. PCoA plots on the Bray-Curtis distance matrices showed that the samples of the breeding season and non-breeding season clustered separately (Adonis tests, *p* < 0.01). The first principal axis could explain 25.24% of the total sample differences, and the second principal axis could explain 12.46% of the sample differences, indicating a possible effect of seasonal breeding strategy (Figure 1).

### 3.3. Taxonomic Composition of the Microbiota

**Figure 2:**
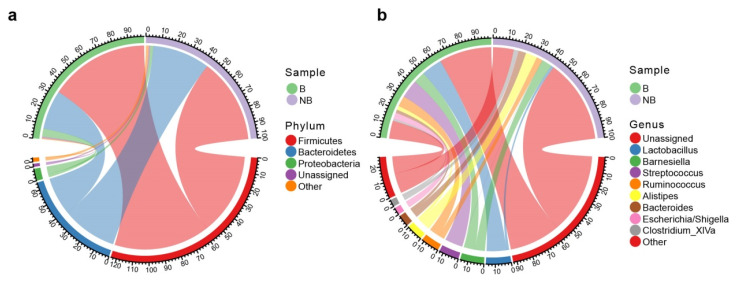
Taxonomic analysis of the gut microbial community composition of each group. Sequences that could not be assigned at the phylum level (**a**) and genus level (**b**) were marked as “Unassigned”. B, breeding season; NB, non-breeding season. (**a**) Chord diagram at the phylum level. (**b**) Chord diagram at the genus level.

Three major bacterial phyla were discovered in the wild ground squirrel gut microbiota, classified as Firmicutes (61.8%), Bacteroidetes (32.4%), and Proteobacteria (3.7%) (Figure 2a and Supplementary Materials Table S3). Other phyla were Verrucomicrobia, Elusimicrobia, Actinobacteria, and Tenericutes, but they were not abundant (<1%). The rest of the sequences of the wild ground squirrel gut microbiota were not annotated and were together called the unassigned group.

At the genus level, being in the unassigned group also means that the sequences cannot be compared to any known genus. The proportion of this unassigned group at the genus level was 46.2% in all samples. The top 10 genera were listed in Figure 2b; the top 4 genera in all samples were Lactobacillus (7.9%), Barnesiella (7.4%), Streptococcus (6.3%), and Ruminococcus (5.7%) (Supplementary Materials Table S3).

### 3.4. Analysis of the Microbiota Difference between the Two Groups

**Figure 3:**
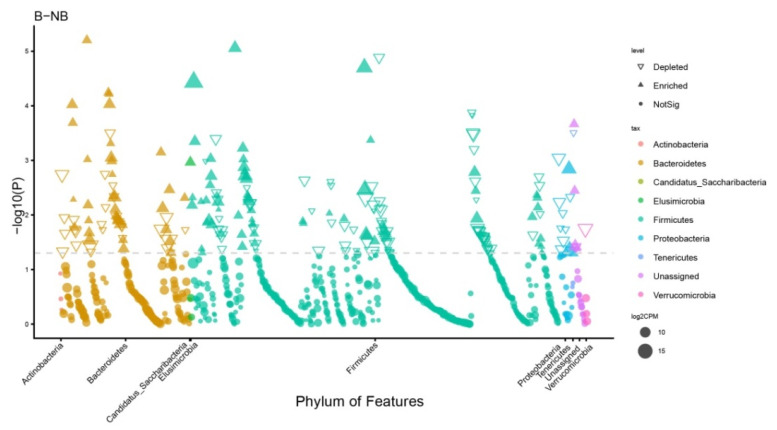
Manhattan plot of the different microbiota at the phylum level. The group of the non-breeding season (NB) was used as the control, and the group of the breeding season (B) was compared with it. The color of each dot represents different taxonomic of the ASVs at the phylum level, and the size of the dot represents the relative abundance of ASVs, takes log2CPM. CPM counts per million. The shape of the dot indicates the type of change.

A Manhattan plot was used to observe the enrichment of ASVs at the phylum level (Figure 3 and Supplementary Materials Table S4; False Discovery Rate (FDR)-adjusted *p* < 0.05, Wilcoxon rank sum test). In both seasons, ASVs were mainly enriched in Bacteroidetes, Firmicutes, Proteobacteria, Tenericutes, and Verrucomicrobia. Proteobacteria, Verrucomicrobiais, and Tenericutes were reduced in the breeding season relative to the non-breeding season. The abundance of Elusimicrobia was significantly higher in the breeding season than in the non-breeding season.

**Figure 4:**
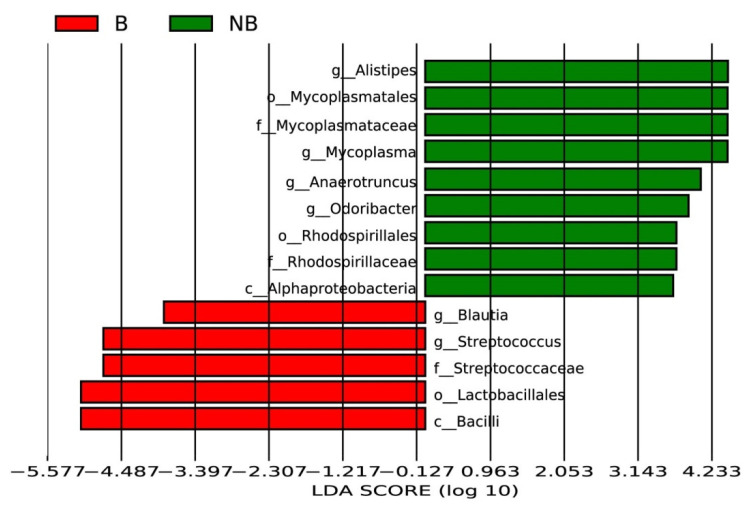
Representative differential microbiota between two groups identified by linear discriminant analysis coupled with effect size (LEfSe) (LDA > 2, *p* < 0.05). Linear discriminant analysis (LDA) is the influence degree of species with significant differences between the two groups. Red box: enriched in the breeding season (B). Green box: enriched in the non-breeding season (NB).

To identify specific microbial communities that exist in the breeding season and non-breeding season, a LEfSe analysis was conducted to explore the microbial community in the two different periods (Figure 4). At the genus level, the wild ground squirrel in the non-breeding season had a significantly higher relative abundance of Alistipes, Mycoplasma, Anaerotruncus, and Odoribacter. In contrast, the wild ground squirrel in the breeding season had higher relative abundance of Blautia and Streptococcus. At the order level, the abundance of Rhodospirillales in the non-breeding season was more than that in the breeding season, and Lactobacillales was more abundant in the breeding season. At the class level, Alphaproteobacteria were significantly enriched in the non-breeding season, and Bacilli were significantly enriched in the breeding season. These results suggest that there are diverse microbial communities in the gut of the wild ground squirrel, and their composition and relative abundance may affect the physiological activities of the host.

### 3.5. Analysis of Functional Differences in the Gut Microbiota between the Two Groups

**Figure 5:**
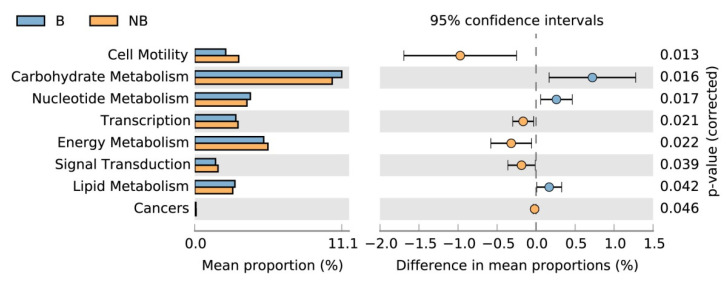
The extended error bar for significant differences in the second level of functional categories of the KEGG pathways. The color of the dots indicates that this pathway is significantly enriched during this period (*p* < 0.05). B, breeding season; NB, non-breeding season.

PICRUSt was used to predict the function of the gut microbiota. The analysis of functional categories of the Kyoto Encyclopedia of Genes and Genomes (KEGG) pathways indicated that the predicted functional categories changed remarkably between the breeding season and the non-breeding season, including cell motility, carbohydrate metabolism, nucleotide metabolism, transcription, energy metabolism, signal transduction, lipid metabolism, and cancers (Figure 5 and Supplementary Materials Table S5). The relative abundance of functional categories that were related to carbohydrate metabolism, nucleotide metabolism, and lipid metabolism was higher in the breeding season than in the non-breeding season. Furthermore, the relative abundance of transcription, energy metabolism, and signal transduction was higher in the non-breeding season than in the breeding season. These results illustrate that the metabolic function of the gut microbiota in the wild ground squirrel is associated with the changes in seasons.

## 4. Discussion

The seasonal breeding of animals is a phenomenon whereby reproduction only occurs in a specific season. In this study, for the first time, we used next-generation sequencing technology to explore the composition of the gut microbiota in the wild ground squirrel. Meanwhile, we demonstrated seasonal changes in the taxonomy and function of the gut microbiota in the wild ground squirrel. Diet, phylogeny, age, genotype, and other factors can affect the composition of the host gut microbiota [40,41,42,43]. Among them, diet is considered to be the most important factor [44]. Our study revealed that there was no significant difference in the diversity and richness of the gut microbiota between the breeding season and the non-breeding season in the wild ground squirrel. This may be due to the relatively free foraging environment in the wild. Based on the Bray-Curtis distance matrices, the PCoA showed that the community structure of the wild ground squirrel gut microbiota clustered separately by breeding season, with differences in community structure among samples from the breeding season and non-breeding season.

The analysis of the gut microbiota indicated that the main bacteria in both the breeding season and non-breeding season for the wild ground squirrel belonged to the phyla Firmicutes and Bacteroidetes, which are also the dominant microbiota species of the mammalian gut [45]. These results are consistent with studies on the gut microbiome of other *Spermophilus* mammals, including arctic ground squirrels (*Urocitellus parryii*) [46] and 13-lined ground squirrels (*Ictidomys tridecemlineatus*) [47]. Firmicutes can degrade polysaccharides through amyloplast and cellulosome multi-enzyme complexes [48] and has positive effects on nutrient and energy absorption from food [49]. Bacteroides can not only degrade polysaccharides, carbohydrates, and proteins in the gut but can also assemble polysaccharides aiding in host nutrient absorption from the diet [48,50]. Moreover, it can also improve the gut environment, being more beneficial to itself and other microorganisms [48]. At the same time, there is a mutualism between Firmicutes and Bacteroides. The high abundance of Firmicutes and Bacteroides has the potential to help the host absorb or store energy, and the higher ratio of Firmicutes to Bacteroides is also related to the prevalence of some diseases, most typically obesity due to increased efficiency of energy harvesting [51,52]. At the genus level, Lactobacillus has the largest proportion in the wild ground squirrel, which is similar to other rodent mammals such as wild wood mice (*Apodemus sylvaticus*) [53] and Siberian hamsters (*Phodopus sungorus*) [54]. These results also indicate that, in the evolution of the vertebrate gut microbiota, the species of bacteria that can settle in the gut are stable [55].

Manhattan analysis showed that the wild ground squirrel of the breeding season and non-breeding season had four different representative communities at the phyla level, including Proteobacteria, Tenericutes, Verrucomicrobia, and Elusimicrobia. Proteobacteria have versatile physiology and greatly variable morphology and are the most unstable phylum compared with the other three major phyla (Firmicutes, Bacteroidetes, and Actinobacteria) [56]. According to the rRNA sequences, the phylum can be divided into six classes: Alpha-, Beta-, Gamma-, Delta-, Epsilon-, and Zetaproteobacteria. It can adjust the metabolism flexibly and tolerate low nutritional food [57]. At the same time, the growth of Proteobacteria can cause biological diseases, and so can be applied as a diagnostic marker of potential disease risk [58]. Our study showed that the phylum of Proteobacteria, the class of Alphaproteobacteria, and the order of Rhodospirillales increased in the gut microbiota of the wild ground squirrel during the non-breeding season. Proteobacteria, which have high abundance in the non-breeding season, may help to improve the digestion and absorption efficiency of the wild ground squirrel, and adjust its metabolism to balance its consumption during the breeding period. Tenericutes are a special class of bacteria that are famous for lacking cell walls, consisting of the sole class Mollicutes [59]. The most significant genus in the phylum Tenericutes is the *Mycoplasma* (Mycoplasmataceae, Mycoplasmatales) [60]. In humans, *Mycoplasma* infection can cause some reproductive disorder diseases, such as infertility [61,62]. Our study illustrated that the relative abundance of *Mycoplasma* in the non-breeding season was significantly increased, which indicates that individuals may be selected for more reproductively suitable taxa during the breeding season to create suitable conditions for reproduction. During the breeding season, the abundance of order Lactobacillales, which belongs to the class Bacilli, increased greatly. Although Lactobacillales, one of the diverse and phylogenetically heterogeneous orders of lactic acid bacteria, are not the most abundant microbiota in the gut, they play an important role in animal reproduction. They can use carbohydrates through fermentation to produce lactic acid, and have a bearing on vitamin B9 (folate) biosynthesis in humans [63,64]. Folate is mainly involved in the synthesis of genetic material and is essential for cell growth and reproduction [65]. Most importantly, Lactobacillales can also indirectly regulate the HPG axis through the activation of leptin [3]. Therefore, the increase of Lactobacillales in the wild ground squirrel during the breeding season is conducive to the improvement of reproductive performance.

In this study, we also used PICRUSt to predict the potential function of the gut microbiota and identified the functional pathways that were differentially enriched between the breeding season and non-breeding season. The mean weighted nearest sequenced taxon index (NSTI) for our samples was 0.129 ± 0.048. This result was similar to previously reported analyses in mammalian gut samples (mean NSTI = 0.14 ± 0.06) [37]. Our results showed that three KEGG pathways (carbohydrate metabolism, nucleotide metabolism, and lipid metabolism) were enriched in the breeding season, and five KEGG pathways (cell motility, transcription, energy metabolism, signal transduction, and cancers) were enriched in the non-breeding season. It has been reported that the gut microbiota plays a significant role in various metabolic processes in the host, including glucose metabolism, lipid metabolism, and energy homeostasis [66]. In view of lipid metabolism, the gut microbiota may affect host lipid metabolism through the metabolites and lipopolysaccharides produced by the gut microbiota [67]. Lipid metabolism affects fetal growth and late pregnancy outcome. Studies in humans and rats have proven that fat pools accelerate breakdown during the last third of pregnancy [68]. Our results indicated that the increase in the lipid metabolism pathway in the wild ground squirrel during the breeding season may be due to increased levels of gut microbiota, which are related to lipid metabolism. This avoids the negative effect of fat accumulation on the body in the breeding season. The function between carbohydrate and lipid metabolism is mutual [69], therefore it is understandable that the carbohydrate metabolism pathway is also enriched in the breeding season. It is speculated that the reason for the enrichment of the energy metabolism pathway in the non-breeding season may be that the host needs to metabolize the energy that is accumulated during the breeding season to maintain its health. These pathways may contribute to the reproduction and homeostasis of the wild ground squirrel.

## 5. Conclusions

In summary, to learn about seasonal changes in the taxonomy and function of gut microbiota of the wild ground squirrel in the breeding season and non-breeding season, 16S rRNA gene sequencing technology was utilized. Difference analyses of LEfSe showed that there was differential enrichment from the phylum to the genus level between the breeding season and non-breeding season. The functional prediction results of PICRUSt showed that the gut microbiota with significant differences mainly existed in metabolic pathways under different reproductive strategies. These results preliminarily revealed the potential association between the gut microbiota and seasonal breeding of animals. The results of this study expand our knowledge of the symbiosis and co-evolution of seasonal breeding animals and their gut microbiota and provide a prerequisite for future studies on the special effects of the gut microbiota on seasonal breeding through a metagenomic analysis.

## Data Availability

The raw data obtained in this paper have been deposited in the Genome Sequence Archive in Beijing Institute of Genomics (BIG) Data Center, Chinese Academy of Sciences (PRJCA004278). The accession number of 16S rRNA gene sequencing data is CRA003793, which can be downloaded from http://bigd.big.ac.cn/gsa (accessed on 3 February 2021). R Scripts and pipeline are employed in the bioinformatic analyses and are available at https://github.com/yuan-zheng-rong/16S-rRNA-analysis-pipeline (accessed on 3 February 2021).

## Supplementary Materials

The following are available online at https://www.mdpi.com/article/10.3390/ani11092685/s1, Figure S1: Alpha diversity rarefaction plots of each group, Figure S2: Alpha diversity of the gut microbiota in the breeding and non-breeding seasons, Table S1: Total reads for each sample before standard quality control (QC) filters. Cleaned sequence length and number of ASVs for each sample after standard quality control (QC) filters, Table S2: The community richness index (ACE and Chao1) and the community diversity index (Shannon and Simpson) for each sample, Table S3: Statistical test of difference analysis in the phylum and the genus level, Table S4: Differential abundance of ASVs between the breeding season and non-breeding season, Table S5: Statistical analysis of KEGG pathways between the two groups.

## Abbreviations

HPG axis	hypothalamus-pituitary-gonadal axis
ERα	estrogen receptor alpha
ERβ	estrogen receptor beta
AR	androgen receptor
P450arom	aromatase cytochrome P450
ASVs	amplicon sequence variants
RDP	Ribosomal Database Project
PCoA	Principal Coordinate Analysis
LEfSe	Linear Discriminant Analysis Effect Size
PICRUSt	Phylogenetic Investigation of Communities by Reconstruction of Unobserved States
BIG	Beijing Institute of Genomics
FDR	false discovery rate
KEGG	Kyoto Encyclopedia of Genes and Genomes
NGS	Next-generation sequencing
NSTI	nearest sequenced taxon index
