# Supplementary material for: Seasonal Changes in the Distinct Taxonomy and Function of the Gut Microbiota in the Wild Ground Squirrel (Spermophilus dauricus)
Source: Animals (Basel). 2021 Sep 13;11(9):2685. doi: 10.3390/ani11092685 (PMC8469230; doi:10.3390/ani11092685)
Supplement: Supplementary file 1 [file animals-11-02685-s001.zip › Figure S2.pdf]

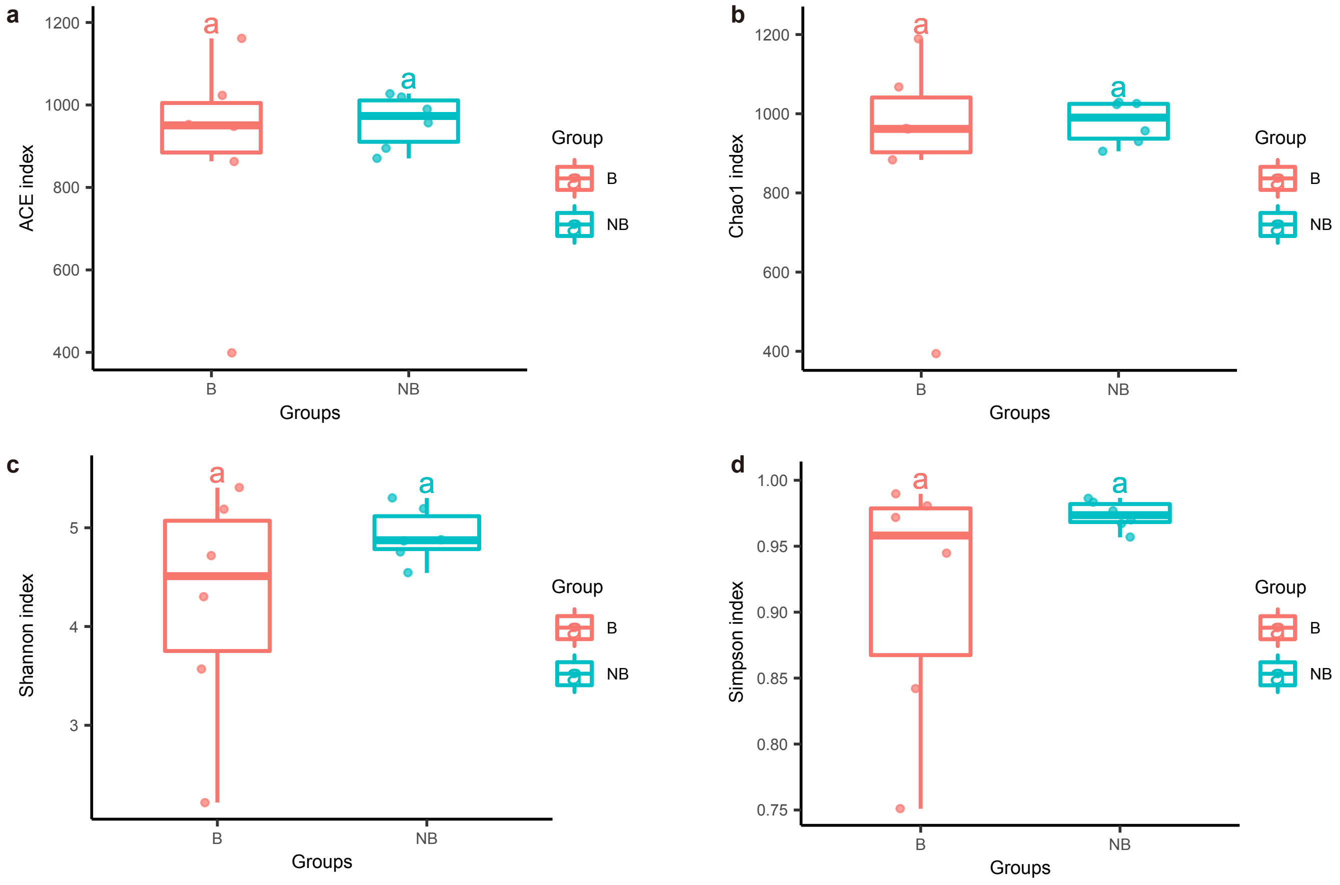

Fig.S2. Alpha diversity of the gut microbiota in the breeding and non-breeding seasons. (a) ACE index (b) Chao1 index (c) Shannon index (d) Simpson index. The top, middle and bottom lines of the boxes represent upper quartiles, medians, and lower quartiles respectively. The upper and lower whiskers extend from the upper and lower edges of the box to data within 1.5× the interquartile range, respectively. B, breeding season; NB, non-breeding season.
