# Supplementary material for: Seasonal Changes in the Distinct Taxonomy and Function of the Gut Microbiota in the Wild Ground Squirrel (Spermophilus dauricus)
Source: Animals (Basel). 2021 Sep 13;11(9):2685. doi: 10.3390/ani11092685 (PMC8469230; doi:10.3390/ani11092685)
Supplement: Supplementary file 1 [file animals-11-02685-s001.zip › Table S1.pdf]

**Table S1.** Total reads for each sample before standard quality control (QC) filters. Cleaned sequence length and number of ASVs for each sample after standard quality control (QC) filters.

| Sample | Total reads | Clean_seqs | ASV counts |
|---|---|---|---|
| B1 | 160966 | 55517 | 1113 |
| B2 | 119978 | 41929 | 865 |
| B3 | 166448 | 56164 | 1026 |
| B4 | 186354 | 61881 | 898 |
| B5 | 180714 | 59584 | 863 |
| B6 | 130630 | 31685 | 317 |
| NB1 | 100274 | 26827 | 204 |
| NB2 | 148044 | 52671 | 255 |
| NB3 | 183978 | 62806 | 209 |
| NB4 | 138644 | 47411 | 232 |
| NB5 | 163972 | 55310 | 241 |
| NB6 | 132296 | 43796 | 203 |

B, breeding season; NB, non-breeding season.
