# Supplementary material for: Seasonal Changes in the Distinct Taxonomy and Function of the Gut Microbiota in the Wild Ground Squirrel (Spermophilus dauricus)
Source: Animals (Basel). 2021 Sep 13;11(9):2685. doi: 10.3390/ani11092685 (PMC8469230; doi:10.3390/ani11092685)
Supplement: Supplementary file 1 [file animals-11-02685-s001.zip › Table S2.pdf]

Table S2. The community richness index (ACE and Chao1), community diversity index (Shannon and Simpson) for each sample.

SampleID	Chao1	ACE	Shannon	Simpson
B1	1189.168	1161.4	5.188146	0.980939
B2	960.5446	952.2633	4.300073	0.944246
B3	1067.103	1022.224	5.406034	0.989723
B4	962.8922	948.7459	3.569155	0.841802
B5	883.2471	863.3297	4.720447	0.971907
B6	393.6512	397.6003	2.217882	0.750912
NB1	1025.778	1027.65	4.867893	0.967579
NB2	1022.585	989.4314	5.301287	0.98375
NB3	905.1235	870.5195	4.879087	0.970492
NB4	957.8264	957.1833	4.542708	0.956739
NB5	1029.827	1018.301	5.195961	0.986655
NB6	930.1932	895.0828	4.755879	0.976622

B, breeding season; NB, non-breeding season.
