# Supplementary material for: Seasonal Changes in the Distinct Taxonomy and Function of the Gut Microbiota in the Wild Ground Squirrel (Spermophilus dauricus)
Source: Animals (Basel). 2021 Sep 13;11(9):2685. doi: 10.3390/ani11092685 (PMC8469230; doi:10.3390/ani11092685)
Supplement: Supplementary file 1 [file animals-11-02685-s001.zip › Table S5.pdf]

Table S5. Statistical analysis of KEGG pathways between the two groups.

Function pathways	B: mean rel. freq. (%)	B: std. dev. (%)	NB: mean rel. freq. (%)	NB: std. dev. (%)	p-values (corrected)	Difference between means	95.0% lower CI	95.0% upper CI
Cell Motility	2.331	0.486	3.303	0.536	0.013	-0.972	-1.694	-0.250
Carbohydrate Metabolism	11.054	0.345	10.332	0.433	0.016	0.722	0.167	1.277
Nucleotide Metabolism	4.183	0.168	3.922	0.103	0.017	0.261	0.059	0.463
Transcription	3.087	0.109	3.252	0.073	0.021	-0.165	-0.299	-0.032
Energy Metabolism	5.185	0.205	5.503	0.161	0.022	-0.319	-0.580	-0.057
Signal Transduction	1.562	0.115	1.749	0.133	0.039	-0.186	-0.362	-0.011
Lipid Metabolism	3.023	0.108	2.855	0.119	0.042	0.168	0.008	0.329
Cancers	0.083	0.016	0.102	0.007	0.046	-0.019	-0.037	0.000

B, breeding season; NB, non-breeding season; mean rel. freq., mean relative frequency; std. dev., Standard Deviation; CI, confidence interval.
