# Supplementary figures and images for: Seasonal Changes in the Distinct Taxonomy and Function of the Gut Microbiota in the Wild Ground Squirrel (Spermophilus dauricus)
Source: Animals (Basel). 2021 Sep 13;11(9):2685. doi: 10.3390/ani11092685 (PMC8469230; doi:10.3390/ani11092685)

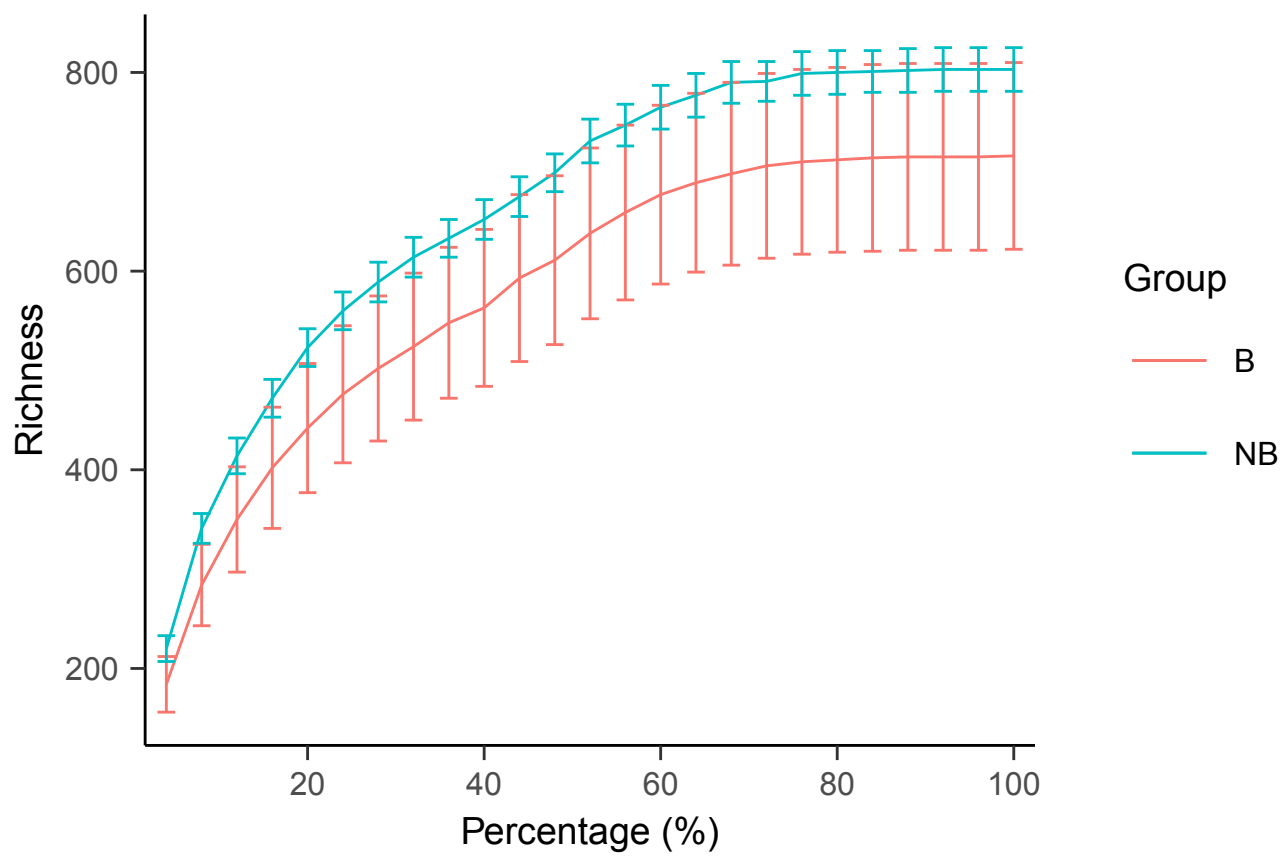

**Fig. S1.** Alpha diversity rarefaction plots of each group. B, breeding season; NB, non-breeding season.

Supplement: Supplementary file 1 [file animals-11-02685-s001.zip › Figure S1.pdf]
